# Automatic Evidence Retrieval for Systematic Reviews

**DOI:** 10.2196/jmir.3369

**Published:** 2014-10-01

**Authors:** Miew Keen Choong, Filippo Galgani, Adam G Dunn, Guy Tsafnat

**Affiliations:** ^1^Centre for Health InformaticsAustralian Institute of Health InnovationUniversity of New South WalesKensington NSWAustralia

**Keywords:** evidence-based medicine, medical informatics, information storage and retrieval

## Abstract

**Background:**

Snowballing involves recursively pursuing relevant references cited in the retrieved literature and adding them to the search results. Snowballing is an alternative approach to discover additional evidence that was not retrieved through conventional search. Snowballing’s effectiveness makes it best practice in systematic reviews despite being time-consuming and tedious.

**Objective:**

Our goal was to evaluate an automatic method for citation snowballing’s capacity to identify and retrieve the full text and/or abstracts of cited articles.

**Methods:**

Using 20 review articles that contained 949 citations to journal or conference articles, we manually searched Microsoft Academic Search (MAS) and identified 78.0% (740/949) of the cited articles that were present in the database. We compared the performance of the automatic citation snowballing method against the results of this manual search, measuring precision, recall, and F_1_ score.

**Results:**

The automatic method was able to correctly identify 633 (as proportion of included citations: recall=66.7%, F_1_ score=79.3%; as proportion of citations in MAS: recall=85.5%, F_1_ score=91.2%) of citations with high precision (97.7%), and retrieved the full text or abstract for 490 (recall=82.9%, precision=92.1%, F_1_ score=87.3%) of the 633 correctly retrieved citations.

**Conclusions:**

The proposed method for automatic citation snowballing is accurate and is capable of obtaining the full texts or abstracts for a substantial proportion of the scholarly citations in review articles. By automating the process of citation snowballing, it may be possible to reduce the time and effort of common evidence surveillance tasks such as keeping trial registries up to date and conducting systematic reviews.

##  Introduction

Evidence retrieval tasks such as literature reviews and decision support, where recall of all relevant evidence is required, cannot rely on search technology alone due to limitations of keyword searching [1]. This has led to the development of secondary search methods such as citation tracking, called snowballing [2], and citation pearl growing [3,4].

Snowballing involves recursively pursuing relevant references cited in already-retrieved literature and adding them to the search results. Thus, snowballing is not limited to citation information found in bibliographical databases. The technical challenges of snowballing include obtaining the full text of retrieved citations, recognizing citation strings in the text, and retrieving new citations from citation strings. These make snowballing both tedious and time consuming.

Unlike keyword searching, snowballing does not require specific search terms [5], which are variable and inconsistent. Rather, it can be thought of as relying on the accumulation of multiple searches from different publishing authors [6]. Snowballing is a complementary approach to search for discovering additional evidence. Demonstrably, snowballing improves retrieval—for example, case studies using search and forward citation tracking on depression and coronary heart disease have been shown to identify more eligible articles than search alone [6]. A review on checking reference lists to find additional studies for systematic reviews found that citation tracking increased the yield of search results by 2.5-43% [7]. Snowballing is considered best practice and is frequently recommended when conducting systematic reviews [2]. With the rapid increase in the rate of publication, automation of snowballing would significantly reduce the time dedicated to literature search.

We tested an approach to automatic snowballing that uses citation extraction algorithms and scientific search engines to follow the steps of snowballing: (1) extract citation strings from documents, (2) find the citations, (3) fetch the full text of citations, and (4) repeat the process to recursively retrieve more citations. The goal of this study is to test the feasibility of automating each of the subtasks of snowballing.

##  Methods

### Algorithm

With an initial set of at least one paper, portable document format (PDF) and hypertext markup language (HTML) documents are converted to plain text. A modified version of ParsCit [8] (a free and open source reference strings segmentation package) identifies reference sections and segments individual reference strings. We modified the heuristics in ParsCit and changed the restriction by allowing the reference label to be found from the middle (50%) to the end of the text. Each reference is converted to a search engine query by removing short words, numbers, and punctuation. The query results returned from the search engine contain citation information (eg, authors, titles, journal, year, digital object identifiers [DOI]) and often one or more links (uniform resource locator [URL]) to full text. We extracted and followed links to the full text. The source code is available in Multimedia Appendix 1 and [9].

### Data

In the evaluation, we used citations from a set of published English language reviews about neuraminidase inhibitors. The dataset consisted of 152 systematic and non-systematic review articles. We randomly selected a subset of 20 review articles that contained 1057 citations. We excluded references to websites, books, book chapters, newspaper articles, and grey literature, leaving 949 included citations. The properties of the 20 review articles are provided in Multimedia Appendix 2.

### Study Design

We evaluated our algorithm using the proportion of extracted references, the proportion of citations retrieved, and the proportion of abstracts and full texts downloaded. We checked extracted citations manually against the references in the paper. We considered a reference to be correctly extracted only if it contained the entire reference without loss of information. We did allow for minimal extra information, such as white space and citation number but not information that should have been part of another citation string, page footer or manuscript text. The accuracy of the retrieved citations and abstract/full text with the references from the systematic reviews were verified manually. Correctly retrieved articles were counted as true positives. Retrieved articles that are not the ones cited were counted as false positives.

**Figure 1:**
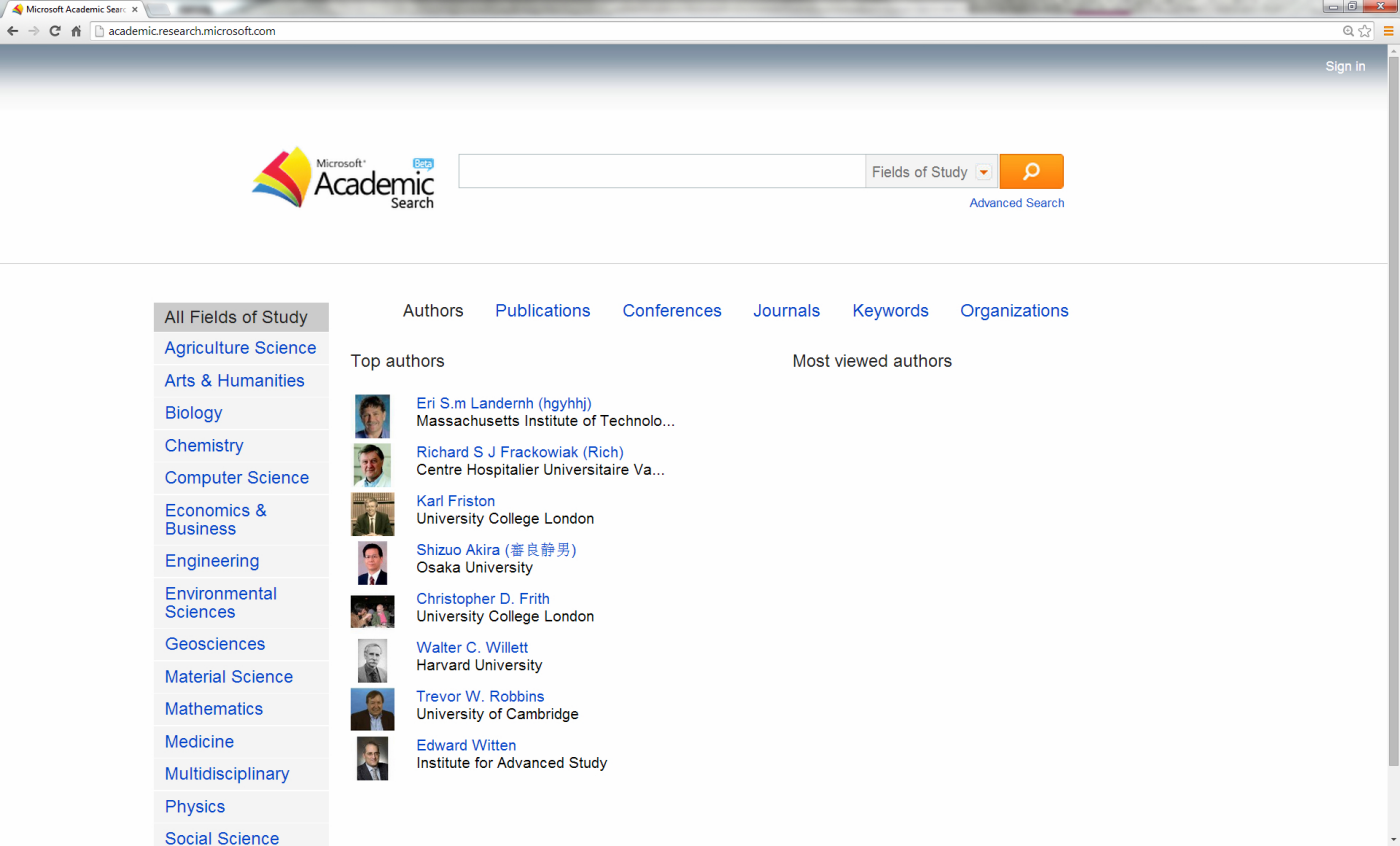
Microsoft Academic Search (MAS).

We used Microsoft Academic Search (MAS) [10] (Figure 1) in the evaluation. MAS is a generalized scientific literature search engine that covers more than 48 million publications with weekly updates. A free application programming interface (API) is provided for non-commercial purposes after registration. Citations include bibliographic data as well as links to citing papers and links to multiple versions of the paper if more than one version exists, including, often, to full text. We used the MAS API to perform searches for each of the references extracted from the full text of the original paper. Other search engines (eg, Google Scholar [11]) can also be used in this step, subject to restrictions they impose. We chose MAS due to its size, “cited by” functionality, links to full text, and because it does not enforce active blocking to prevent automated access.

We manually searched for missed references to ascertain whether they were indeed indexed in MAS. Articles that were not retrieved but were found by manual search of MAS were counted as false negatives. We calculated precision, recall, and F_1_ score using the standard formulae:

Precision = (True positives) / (True positives + False positives)

Recall = (True positives) / (True positives + False negatives)

F_1_ score = 2 x Precision x Recall / (Precision + Recall)

The precision, recall, and F_1_ scores were computed for retrieval of citations, abstract (only abstracts or abstracts with full texts), and full text against all citations (1057 references), included citations (949 references), and included citations indexed in MAS (740 references).

All experiments were conducted on computers with Internet protocols (IP) allocated to the University of New South Wales. Journals that automatically recognize subscription by IP address and to which the University of New South Wales library is subscribed were thus granted access. No other subscription activation or authentication methods were used. However, since most abstracts are freely accessible, download of abstracts will not normally be affected by journal subscription.

##  Results

**Figure 2:**
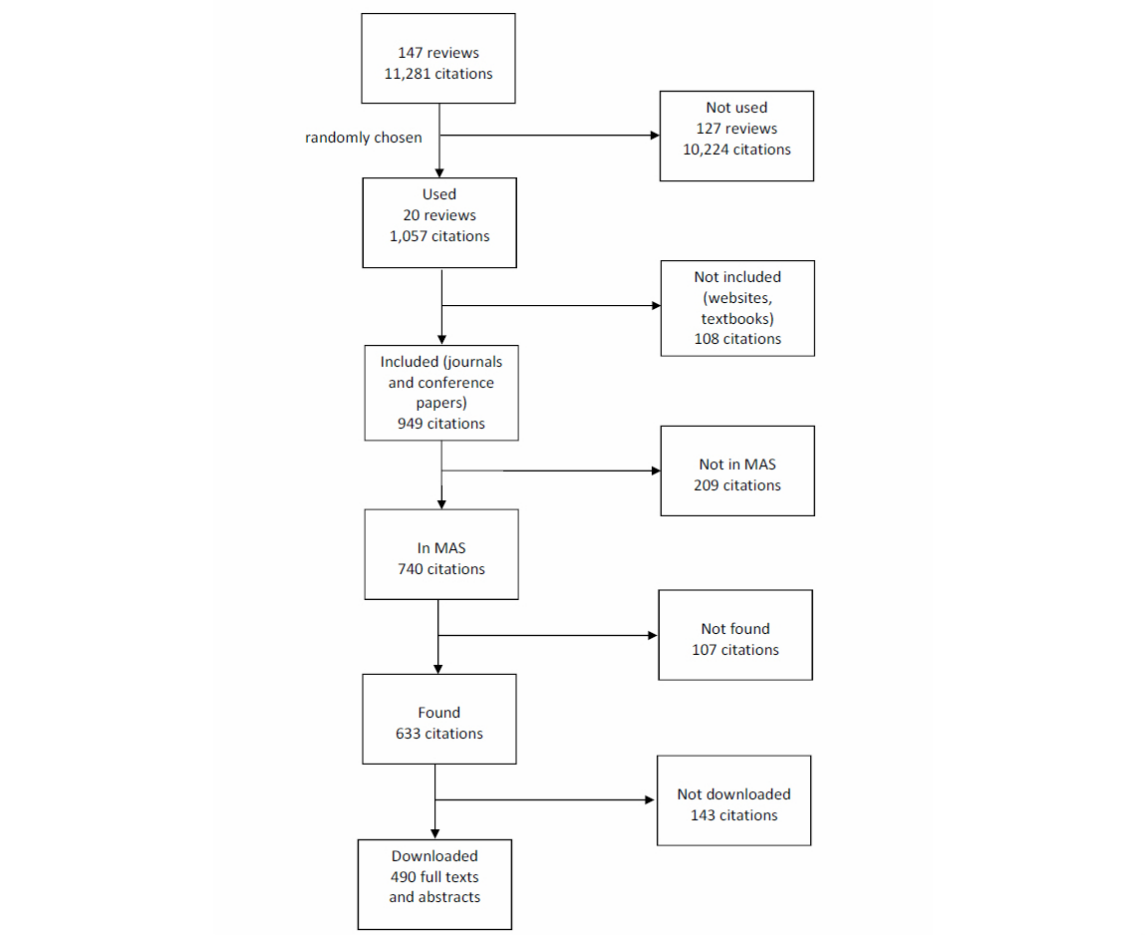
Summary of the evaluation results (from 20 reviews with 949 scholarly citations, MAS included 740 citations, 633 citations were found, and 490 full texts and abstracts were downloaded automatically).

The summary of the evaluation is shown in Figure 2. We successfully extracted 97.2% (922/949) of the included citations (96.5%, 1020/1057 citations) from the 20 reviews randomly selected. The precision, recall and F_1_ scores for retrieval of citations, abstract (only abstracts or abstracts with full texts), and full text fetching of citations from the 20 randomly selected review articles are shown in Table 1. The scores are computed using three categories: (1) all citations, (2) included citations, and (3) included citations indexed in MAS. The first category shows the probability to retrieve a given reference from a review paper. The second category gives the same probability but excludes works not likely to be retrievable such as books and grey literature. The third category excludes citations not currently indexed in MAS.

For the reference strings indexed in MAS, 66.2% (490/740) of abstracts were correctly downloaded either on their own or as part of the full text. These represent 51.6% of 949 included citations and 46.4% of all 1057 references included in the study.

Out of the 633 correctly identified citations, we retrieved the full text or abstract for 490 (recall=82.9%, precision=92.1%, F_1_ score=87.3%). We examined the specific reasons why 143 (22.6%) of the articles were not downloaded automatically. MAS had incorrect links for 39 articles (6.2%), and no link to full text for 6 articles (0.9%); 56 links (8.8%) led to a page that uses JavaScript to dynamically create a link to the full text. For citations where only abstracts were downloaded (15 abstracts), full text documents were not downloaded due to journal subscription access.

**Table 1:** Results of citations, abstract, and full text retrieval (precision, recall, and F_1_ score of database results fetch, and full text and abstract retrieval, comparing all reference strings, only included citations, and only included citations indexed in MAS).

		As proportion of all citations (n=1057)	As proportion of included citations (n=949)	As proportion of citations in MAS (n=740)
**Citations retrieved**				
	Precision	0.977	0.977	0.977
	Recall	0.600	0.667	0.855
	F_1_score	0.743	0.793	0.912
**Abstracts fetched**				
	Precision	0.921	0.921	0.921
	Recall	0.483	0.540	0.702
	F_1_score	0.634	0.681	0.797
**Full text fetched**				
	Precision	0.919	0.919	0.919
	Recall	0.475	0.533	0.696
	F_1_score	0.626	0.674	0.792

##  Discussion

### Principal Findings

Snowballing is tedious and resource demanding but has shown to improve retrieval. This evaluation shows that it is feasible to automatically perform snowballing using our method by extracting and downloading the citations. Systems designed to perform many of the systematic review tasks are already in use, in development, or in research [12,13]. This study represents a first effort to automate the snowballing tasks in a systematic review process. When integrated with a reliable automatic screening tool, automatic snowballing can have a compound effect and increase recall [12].

Automatic citation extraction is a difficult task [14], which causes the citation retrieval to be an even harder task. However, if unique identifier of citations (eg, DOI or PubMed identifier) is provided for each citation, this would greatly improve the reliability of citation extraction and retrieval.

### Limitations

A limitation of this study is that full text fetching is tested on journal subscription by IP address and to which the University of New South Wales library is subscribed. While this means that results may vary in other institutions, they also represent an exemplar that may guide expectations of results. With the growth of open source and other means of obtaining full text [15], the performance of our algorithm may improve.

In this evaluation, the algorithm was limited to MAS. This is a constraint of the testing system, not of the method. From the limited testing we have conducted, the algorithm performs equivalently on Google Scholar but computer-access restrictions prevented a robust comparison.

Some existing databases, such as Scopus [16] and Web of Science [17] (subscription fees apply for both), provide citation analysis and allow one to search both forward (references cited in an investigated text) and backward (papers citing an investigated text) and can thus aid manual snowballing. However, those citations are limited to papers indexed in the respective database. Our method automatically extracts citations directly from documents and can thus cross database boundaries.

### Conclusions

Snowballing is automatable and can reduce the time and effort of evidence retrieval. It is possible to reliably extracts reference lists from the text of scientific papers, find these citations in scientific search engines, and fetch the full text and/or abstract.

## Multimedia Appendix 1

Source code http://www2.chi.unsw.edu.au/~miewkeen/ESuRFr.html.

## Multimedia Appendix 2

Properties of the 20 review articles included in the study.

## Abbreviations

API: application programming interface
DOI: digital object identifier
IP: Internet protocol
MAS: Microsoft Academic Search
