# Supplementary material for: Automatic Evidence Retrieval for Systematic Reviews
Source: J Med Internet Res. 2014 Oct 1;16(10):e223. doi: 10.2196/jmir.3369 (PMC4211030; doi:10.2196/jmir.3369)
Supplement: Supplementary file 1 [file jmir_v16i10e223_app1.zip › EsuRFr/input/009_PMID_16303549.pdf]

8. Nielsen, R., Bustamante, C., Clark, A.G., Glanowski, S., Sackton, T.B., Hubisz, M.J., Fledel-Alon, A., Tanenbaum, D.M., Civello, D., White, T.J., *et al*. (2005). A scan for positively selected genes in the genomes of human and chimpanzee. PLoS Biol. *3*, e170.
9. King, M.C., and Wilson, A.C. (1975). Evolution at two levels in humans and chimpanzees. Science *188*, 107–116.
10. Keightley, P.D., Lercher, M.J., and Eyre-Walker, A. (2005). Evidence for widespread degradation of gene control regions in hominid genomes. PLoS Biol. *3*, e42.
11. Enard, W., Fassbender, A., Model, F., Adorjan, P., Pääbo, S., and Olek, A. (2004). Differences in DNA methylation pattens between humans and chimpanzees. Curr. Biol. *14*, R148–R149.
12. Khaitovich, P., Weiss, G., Lachmann, M., Hellmann, I., Enard, W., Muetzel, B., Wirkner, U., Ansorge, W., and Pääbo, S. (2004). A neutral model of transcriptome evolution. PLoS Biol. *2*, e132.
13. Khaitovich, P., Hellmann, I., Enard, W., Nowick, K., Leinweber, M., Franz, H., Weiss, G., Lachmann, M., and Pääbo, S. (2005). Parallel patterns of evolution in the genomes and transcriptomes of humans and chimpanzees. Science *309*, 1850–1854.
14. Lercher, M.J., Williams, E.J., and Hurst, L.D. (2001). Local similarity in evolutionary rates extends over whole chromosomes in human-rodent and mouse-rat comparisons: implications for understanding the mechanistic basis of the male mutation bias. Mol. Biol. Evol. *18*, 2032–2039.
15. Hardison, R.C., Roskin, K.M., Yang, S., Diekhans, M., Kent, W.J., Weber, R., Elnitski, L., Li, J., O'Conner, M., Kolbe, D., *et al.* (2003). Covariation in frequencies of substitution, deletion, transposition, and recombination during eutherian evolution. Genome Res. *13*, 13–26.
16. Haldane, J.B.S. (1947). The mutation rate of the gene for hemophilia and its segregation ratios in males and females. Ann. Eugen. *13*, 262–271.
17. Hwang, D.G., and Green, P. (2004). Bayesian Markov chain Monte Carlo sequence analysis reveals varying neutral substitution patterns in mammalian evolution. Proc. Natl. Acad. Sci. USA *101*, 13994–14001.
18. Lercher, M.J., and Hurst, L.D. (2002). Human SNP variability and mutation rate are higher in regions of high recombination. Trends Genet. *18*, 337–340.
19. Meunier, J., and Duret, L. (2004). Recombination drives the evolution of GC-content in the human genome. Mol. Biol. Evol. *21*, 984–990.
20. Smith, N.G., Webster, M.T., and Ellegren, H. (2002). Deterministic mutation rate variation in the human genome. Genome Res. *12*, 1350–1356.

Department of Evolutionary Biology, Evolutionary Biology Centre, Uppsala University, Norbyvägen 18D, SE-752 36 Uppsala, Sweden.
E-mail: Hans.Ellegren@ebc.uu.se.

DOI: 10.1016/j.cub.2005.10.060

Infectious Disease: Can We Avert a Lethal Flu Pandemic?

If avian flu becomes directly transmissible among humans, could we prevent a pandemic by using prophylactic antivirals? Possibly, if the virus is not too transmissible, and we react fast and efficiently.

Robert M. May

Since 1997, a highly pathogenic avian influenza virus (AIV), of the subtype H5N1, has emerged in poultry in Southeast Asia. Concern is rising, for several reasons. In May 2005, the virus was found in migratory waterfowl in western China [1] and most recently AIV is reported among birds in Turkey and Romania. To date, essentially all the few, but increasing, human cases appear to have come from human-to-bird contact, causing around 60 deaths from something like 120 known infections [2]. Past flu pandemics are believed to have arisen when AIV has reassorted with a human influenza virus, within a dually infected human patient, resulting in a new variant capable of direct transmission from human to human.

So, what is our chance of containing such a H5N1 or similar flu epidemic, if and when it appears? This question is addressed in two separate and recent numerical studies [3,4]. Both assume that AIV jumps to humans in Southeast Asia, and use evidence from experience with previous flu epidemics to assess the parameters in complex and detailed models of the spread of infection originating in Thailand, and the possibilities of averting a pandemic.

Central to any such study is the infection's basic reproductive number, R_0, which quantifies its transmissibility; R_0 is defined as the average number of secondary cases generated by a typical primary case in an entirely susceptible population [5]. Epidemics can arise if R_0 exceeds one, and not otherwise. Control strategies aim to reduce R_0 below one, by effectively removing a proportion $1 - (1/R_0)$ of the susceptible population.

Longini *et al*. [3] estimate R_0 as around 1.4 — based on about 33% of the population being infected in past Asian pandemics — but their simulations explore R_0-values from 1.1 to 2.4. Ferguson *et al*. [4] estimate R_0 at 1.8 — based on re-analysis of earlier data, suggesting the average 'generation interval' between an individual becoming infected and infecting a contact is around 2.6 days, rather than the previously estimated 4 — but they also explore a range $1 < R_0 < 2$. Longini *et al*. [3] assume a model population of 500,000 people, with age-structure and patterns of movement within and between 36 geographical regions, characteristic of rural Thailand (as revealed in its 2000 census). Ferguson *et al*. [4], using massive parallel computational capacity, work with spatially detailed simulations of the 85 million people in Thailand and in a 100 kilometre-wide zone outside its borders, explicitly including households, schools and workplaces.

Figure 1 illustrates how an outbreak of flu might spread in Thailand, if there is no intervention, roughly 90 days after the first case. It comes from Ferguson *et al*.'s [4] simulations, seeded with a single rural individual. For the first 30 days, the epidemic tends to be spatially confined. As 'sparks' are increasingly shed into other regions, numbers increase exponentially and infectious individuals spread over larger distances [6]. Between 60 and 90 days the epidemic changes from being mainly local to being country-wide. Any control strategy needs to be

implemented effectively before this time; after this, logistic constraints make success unlikely. If R_0 is 1.8, the unchecked epidemic infects roughly two-thirds of the modelled population of 85 million.

Methods available for stopping a flu epidemic come under three headings. First, ‘targeted antiviral prophylaxis’ (TAP). Antiviral drugs, of which the most effective currently is oseltamivir (Trade Name ‘Tamiflu’, which inhibits the action of neuraminidase, the N of H5N1), work by significantly reducing transmission. Although they can also help an individual to fight off infection, for population-level prophylaxis they need to be administered in advance of apparent infection. Ferguson *et al*. [4] estimate that blanketing an entire country or region with Tamiflu should be able to eliminate a pandemic virus with an R_0 of even 3.6 or greater. But such action is logistically difficult, if not impossible. Targeted strategies are therefore needed.

Both Ferguson *et al*. [4] and Longini *et al*. [3] look at forms of social targeting as the most straightforward approach. This involves prophylaxing individuals in the same household, school or workplace as newly diagnosed symptomatic cases. And doing so very promptly. Indeed, given that ‘social targeting’ may be too slow to be effective, both sets of authors assume somewhat wider targeting to local neighbourhoods. Longini *et al*. [3] call this Geographical targeted antiviral prophylaxis (GTAP). Second, vaccination would be excellent if we had a vaccine. Although a human influenza H5N1 vaccine is currently being tested, and may be available in time, the problem is — as is the case for normal seasonal flu vaccines — we cannot be certain how effective the vaccine under development may be against the strain that eventually appears. Third, we have quarantine, or other ways of effectively reducing contact rates within the population (Ferguson *et al*. [4] call this “social distance measures”).

Exploring various combinations of these actions, Longini *et al*. [3] concluded that a prepared response with GTAP would have a high probability of containing the epidemic, provided R_0 was below 1.6. Such containment is achieved, in their simulations, with an antiviral stock pile of around 100,000 to 1 million courses of treatment. If effective pre-vaccination occurred — assuming only 30% efficiency, and only halving transmissibility — then TAP could be effective against strains with R_0 as high as 2.1. And if one could use combinations of TAP, pre-vaccination and quarantine, then success could be achieved even against strains with R_0 as high as 2.4.

Ferguson *et al*.’s [4] studies of control were focussed mainly on targeted antiviral prophylaxis. This was partly because they saw logistic difficulties in “social distance measures”, and partly because they believed some such actions could have unintended adverse consequences as a result of people dispersing. Broadly, their more computationally ambitious model — remember, they included the actual 85 million people in and around Thailand, versus Longini *et al*.’s [3] 500,000, rather tidily distributed — concluded that a combination of GTAP and social distance measures could contain an outbreak in Thailand and avoid a pandemic, provided the new virus’ R_0 is below 1.8. But their corresponding calculations required a stockpile of 3 million courses of antiviral drugs. And for larger values of R_0, even this would seem insufficient.

In the light of these conclusions, a glance backward to 1918 is in order. The number killed in that pandemic is estimated at 20 to 50 million. But the global population then was less than 2 billion, with only one quarter urban. And the relatively smaller number of people crossing oceans did so in ships. Today’s threatened pandemic looms over a more crowded world of 6.5 billion, half urban, constantly and rapidly moving around. We know much more, but our circumstances are inherently more difficult.

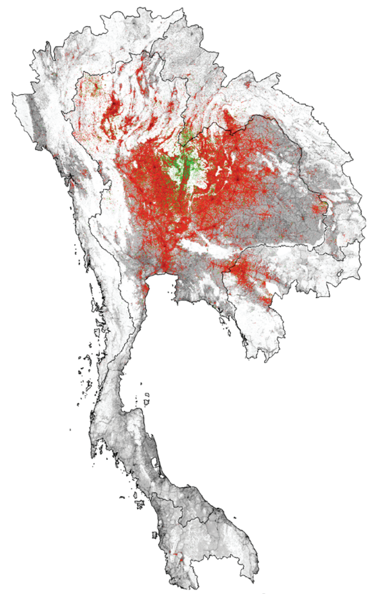

Current Biology

Figure 1. Simulated state after 90 days of a flu epidemic in Thailand, seeded by a single rural individual.

Red, density of infected individuals; green, density of people who have recovered from infection or died; grey, density of uninfected population. (Courtesy of Neil Ferguson; reproduced with permission from [4]).

In the UK, the government has announced plans to stockpile enough courses of Tamiflu to cover one quarter of the UK population by the end of 2006. In a world of good surveillance, efficient delivery of TAP, and no panic, this would be adequate provided H5N1 waits until the UK is ready. In the real world of panicking people, the UK plans could prove more problematic. Meanwhile, the USA is committing $25 million to boosting surveillance in Asia. An official at the National Immunization Program of the US Centers for Disease Control has juxtaposed this sum against the $800 million spent by the USA on anthrax vaccines, “against a pathogen that has killed only a handful of Americans and whose bioterrorist potential is unproven” [7]. We live in interesting times.

References

1. Liu, J., Xiao, H., Lei, F., Zhu, Q., Qin, K., Zhang, X.W., Zhang, X.L., Zhao, D., Wang, G., Feng, Y., *et al.* (2005). Highly pathogenic H5N1 influenza virus

infection in migratory birds. Science *309*, 1206.
2. WHO. (2005). Cumulative number of confirmed human cases of Avian Influenza. http://www.who.int/csr/disease/avian_influenza/table_2005_10_10/en/... 16/10/2005.
3. Longini, I.M., Jr., Nizam, A., Xu, S., Ungchusak, K., Hanshaoworakul, W., Cummings, D.A., and Halloran, M.E. (2005). Containing pandemic influenza at the source. Science *309*, 1083–1087.
4. Ferguson, N.M., Cummings, D.A., Cauchemez, S., Fraser, C., Riley, S., Meeyai, A., Iamsirithaworn, S., and Burke, D.S. (2005). Strategies for containing an emerging influenza pandemic in Southeast Asia. Nature *437*, 209–214.
5. Anderson, R.M., and May, R.M. (1991). Infectious Diseases of Humans: Dynamics and Control (Oxford: Oxford University Press).
6. May, R.M., Gupta, S., and McLean, A.R. (2001). Infectious disease dynamics: what characterizes a successful invader? Phil. Trans. R. Soc. Lond. B Biol. Sci. *356*, 901–910.
7. Butler, D. (2005). Drugs could head off a flu pandemic – but only if we respond fast enough. Nature *436*, 614–615.

Zoology Department, Oxford University, Oxford OX1 3PS, UK.

DOI: 10.1016/j.cub.2005.10.063

Eye Development: Stable Cell Fate Decisions in Insect Colour Vision

The retina provides an example of a fundamental property of developing cells: cell fate decisions are stable. A recent paper reports a double negative feedback loop that leads to bistable fate decisions in the colour-detecting photoreceptors of *Drosophila*.

Matthew Freeman

There is no place in an animal for a cell with an identity crisis. A confused cell is unlikely to function properly and may be dangerous if, for example, it starts to proliferate inappropriately. The concept that cells need to make stable, all-or-nothing fate decisions was developed by Waddington in the first half of the last century [1]. He termed this process ‘canalisation’ and illustrated the idea with artistic drawings of what he called an epigenetic landscape (Figure 1A). His rather theoretical idea has proved to be correct and fundamental. It is becoming apparent that a variety of mechanisms exist to ensure that developmental decisions are robust [2–4]. Although most studies have focussed on the ability of chromatin to convert transient transcriptional states, triggered by apparently fleeting developmental signals, into long-term cellular memory, the increasingly detailed knowledge of signalling mechanisms has led to a recognition that these pathways themselves have sometimes evolved into robust networks that generate stable decisions [5].

An example of apparently similar cells making distinct fate decisions are the colour-detecting photoreceptors of the retina. To see colours, our brain computes the outputs of photoreceptors with different spectral sensitivities. The fruitfly *Drosophila* has a random mosaic of colour-detecting photoreceptors in its retina (Figure 1B) [6] and a recent paper [7] has described a mechanism that ensures that these cells make a robust choice between alternative colour-detecting fates. The *Drosophila* compound eye is formed from about 800 unit eyes (ommatidia), each with six monochromatic outer photoreceptors surrounding two stacked colour detecting photoreceptors, known as R7 and R8. The R7/R8 pair comes in two randomly distributed forms: 70% are ‘yellow’, with R7 expressing the rhodopsin Rh4 and R8 expressing rhodopsin Rh6; the remaining 30% are ‘pale’, with R7 expressing Rh3 and R8 expressing Rh5 (‘yellow’ and ‘pale’ refer to their appearance under a microscope) [8,9]. Yellow ommatidia detect longer wavelengths, into the green part of the spectrum, while the pale ones detect shorter wavelengths, in the blue and UV range.

The decision between a yellow or pale fate is initiated when the R7 cell in each ommatidium makes an apparently stochastic choice of whether to express Rh3 or Rh4. This decision is then communicated to the adjacent R8 cell, forcing it to comply with the ‘golden rule’ that R8 must express Rh5 if partnered with an Rh3-expressing R7, or Rh6 if partnered with a Rh4-expressing R7 [9] (Figure 2A). To investigate further the mechanism underlying retinal mosaicism, Mikeladze-Dvali *et al*. [7] searched for genes expressed in mosaic patterns and found one that was restricted to yellow R8s. Intriguingly this was the *warts* (*wts*) gene (also known as *lats*), encoding a cytoplasmic serine/threonine kinase already famous for its role as a tumour suppressor that regulates cell growth and death [10]. Their attention was then drawn to

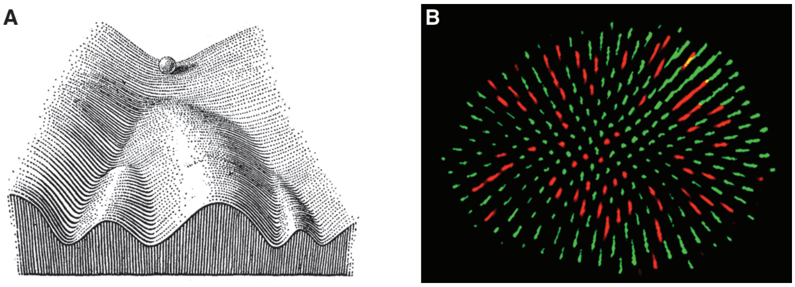

Figure 1. Robustness and cell fates in the *Drosophila* eye.

(A) Waddington’s epigenetic landscape. Cell fates are represented by valleys and, as cells ‘roll down the hill of development’, they are increasingly unable to cross the intervening ridges, i.e. their fates become robust. From [13]. (B) Fluorescent micrograph of a *Drosophila* retina showing the stochastic distribution of ‘yellow’ (labeled in green) and ‘pale’ (red) R8 photoreceptor cells. Image courtesy of Claude Desplan.
