# Supplementary material for: Automatic Evidence Retrieval for Systematic Reviews
Source: J Med Internet Res. 2014 Oct 1;16(10):e223. doi: 10.2196/jmir.3369 (PMC4211030; doi:10.2196/jmir.3369)
Supplement: Supplementary file 2 [file jmir_v16i10e223_app2.pdf]

Appendix 2: Properties of the 20 review articles included in the study.

PubMed ID	No of citations	No of citations extracted with ParsCit	No of scholarly citation (journals/conference papers)	No of scholarly citations manually found in MAS
16303549	7	6	5	5
16443037	71	68	66	36
16503711	63	62	55	44
16651383	63	60	48	40
17414089	75	73	66	54
17483860	40	39	36	33
18044060	36	35	34	27
18157800	24	23	21	19
18664986	50	48	45	35
19067611	35	34	27	22
19797457	16	15	15	13
19802552	34	33	22	19
19935415	71	68	69	54
20008597	20	19	13	6
20085663	49	47	44	36
21406435	49	47	48	38
21673413	25	23	16	15
21850418	34	32	31	26
21964179	202	198	202	156
22371849	93	90	86	62
Total	1057	1020	949	740
